# Next generation sequencing data in the phylogenetic relationships of the genus *Molossus* (Chiroptera, Molossidae)

**DOI:** 10.1016/j.dib.2020.105276

**Published:** 2020-02-14

**Authors:** Livia O. Loureiro, Mark D. Engstrom, Burton K. Lim

**Affiliations:** a Department of Ecology and Evolutionary Biology, University of Toronto, Toronto, ON M5S 3B2, Canada; b Department of Natural History, Royal Ontario Museum, Toronto, ON M5S 2C6, Canada; c The Centre for Applied Genomics, SickKids Hospital, Toronto, ON, M5G 0A4, Canada

**Keywords:** Next generation sequencing, Phylogeny, Molossidae, Evolution, Bats

## Abstract

The mastiff bat *Molossus* is a broadly distributed genus within the family Molossidae. *Molossus* includes groups of species that are either morphologically or genetically very similar, rendering the taxonomy of this genus confusing and unstable. In this paper, we provide inferred phylogenetic relationships of *Molossus* based on the genotype by sequencing approach from 189 specimens of three species of New World mastiff bats (*Molossus*, *Promops*, and *Eumops*). We also present data on divergent tree topologies produced by alignments using *de novo* and reference genome approaches and distinct phylogenetic methods (maximum likelihood and coalescent approaches). These data provide the first highly resolved phylogenetic tree for *Molossus*, not recovered by previous studies using Sanger sequencing. Our dataset brings new insights on relationships among species and show how different approaches might affect phylogenetic resolution and topologies.

**Table:** Specifications Table

Subject	Ecology, Evolution, Behaviour and Systematics
Specific subject area	Genetics, Molecular phylogeny
Type of data	Figures
How data were acquired	We isolated the DNA with the Qiagen DNeasy extraction kit (Qiagen, Inc. Valencia, CA, USA) following the manufacturer's instructions. We sequenced all libraries using the protocol described by Elshire et al. [2] on an Illumina HiSeq 2000.
Data format	Raw and analyzed
Parameters for data collection	We quantified the total DNA using a Nanodrop spectrophotometer (Nanodrop Technologies) and checked the quality by visual inspection on agarose gels. For library preparation, we used thirty microlitres of high quality (>100 ng/ul) DNA per individual.
Description of data collection	We used the Universal Network-Enabled Analysis Kit (UNEAK) and the Discovery pipelines, available as part of the TASSEL 3.0 software [3] to convert raw sequence files from Illumina into individual genotypes. We used the Singular Value Decomposition Scores for Species Quartets (SVDquartets) coalescent approach [4] implemented in PAUP 4.0 and the Maximum Likelihood approach (ML) implemented in FastTree [5] to access phylogenetic relationships.
Data source location	Appendix 1
Data accessibility	Repository name: Mendeley Data • Data identification number: https://doi.org/10.17632/68v2bs4xr9.1 Direct URL to data: https://data.mendeley.com/
Related research article	Loureiro, L.O., Engstrom, M.D, Lim, B.K. 2020. Single Nucleotide Polymorphisms (SNPs) provide unprecedented resolution of species boundaries, phylogenetic relationships, and genetic diversity in the mastiff bats (*Molossus*). Molecular Phylogenetics and Evolution. 143, 1055-7903

**Table:** 

**Value of the Data** • Sanger data have been insufficient to resolve phylogenetic relationships within *Molossus* due to low rates of nucleotide polymorphism among some taxa [6,7]. The next generation data provided here combined with a large geographic sampling, clarify the evolution of *Molossus* and provide well supported definitions of species boundaries and phylogenetic relationships among species. • These data will be the foundation for further studies on biogeography, population ecology, and evolution of morphological and behavioural traits. • These data bring new insights on differences between assemblies derived *de novo* and using reference genomes and could be used to assess bias in methodologies using either approach.

## 1 Data description

**Fig. 1:**
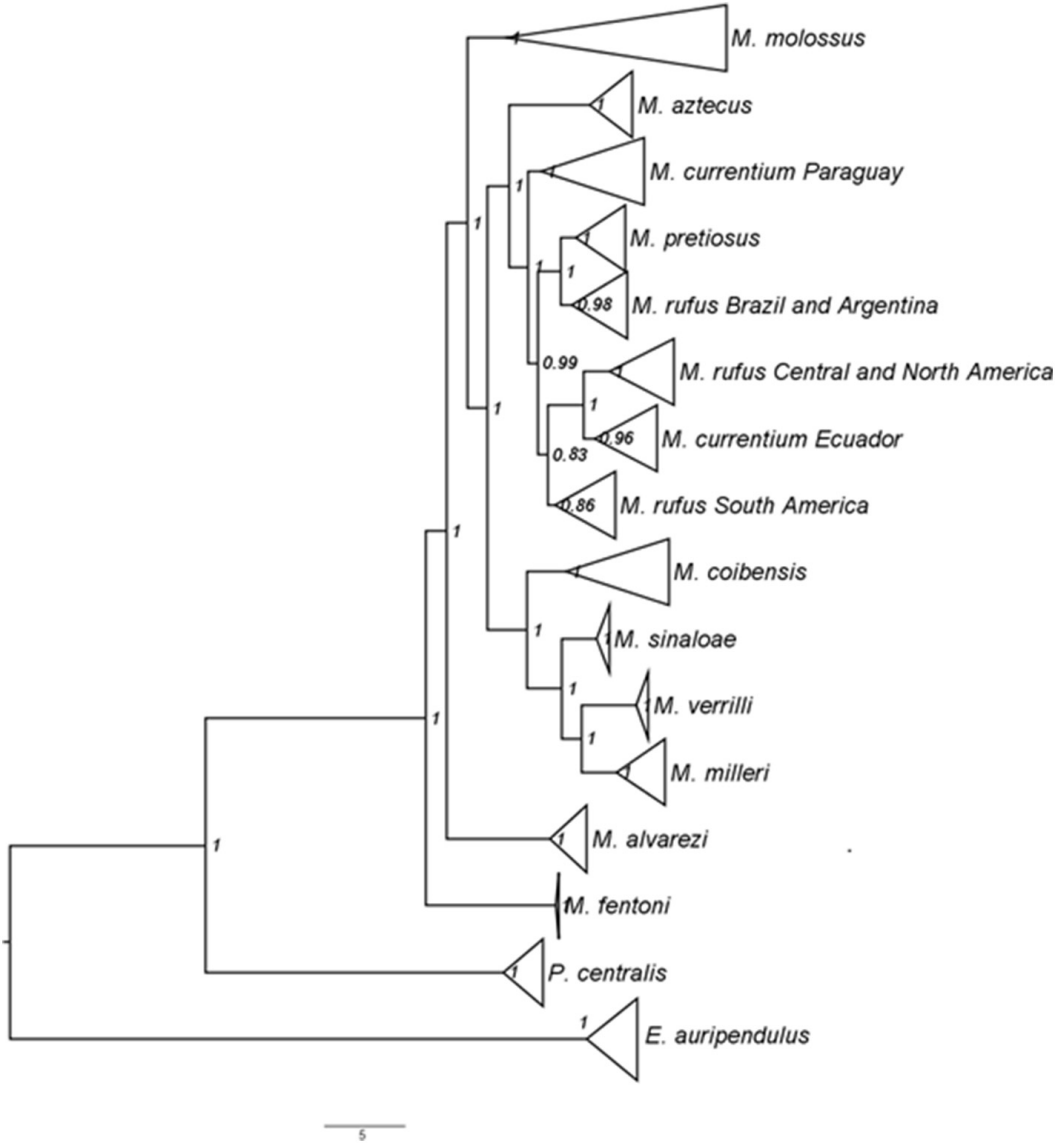
Maximum likelihood tree with the alignment using the *Myotis* reference genome produced by the Discovery pipeline for *Molossus*. Numbers represent bootstrap support values.

**Fig. 2:**
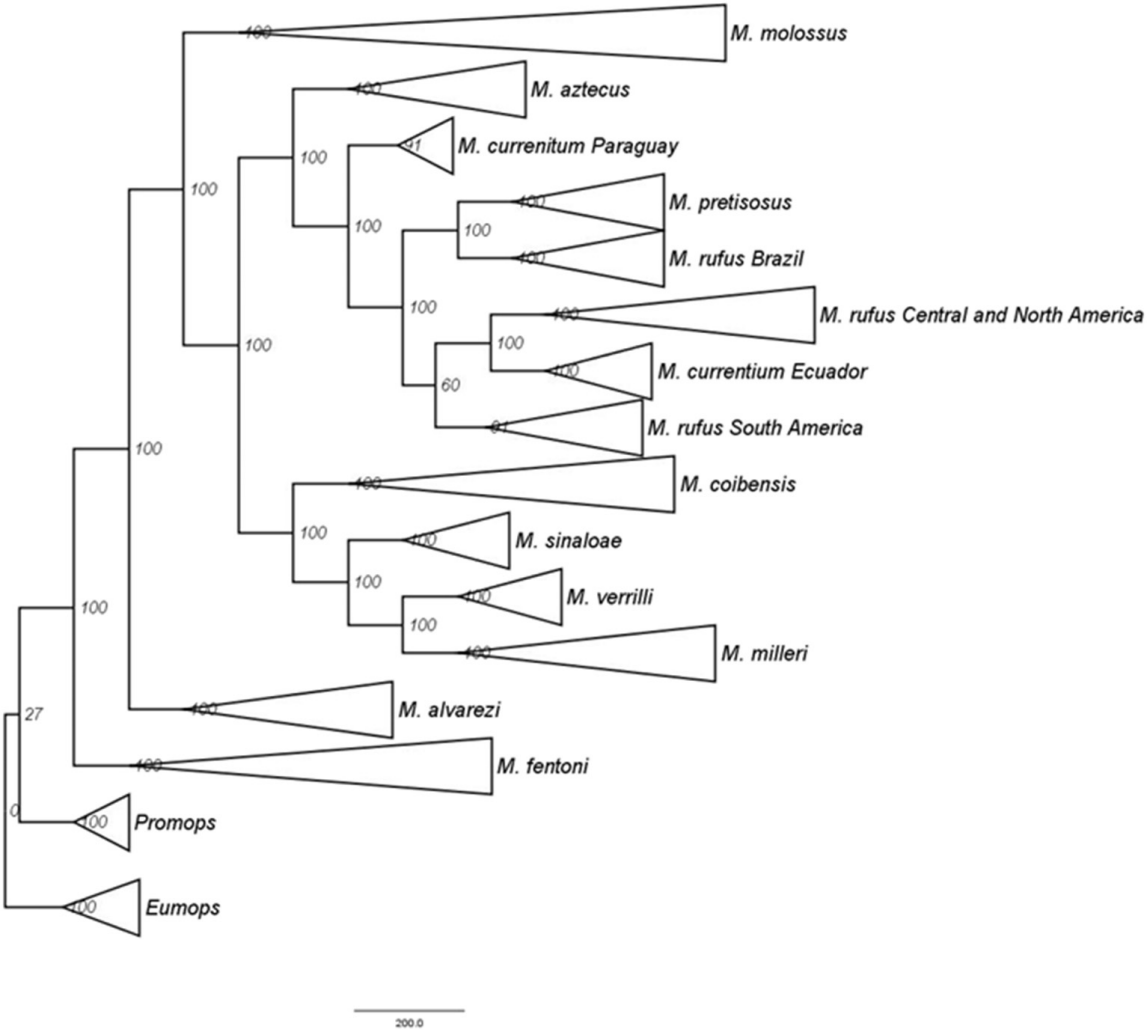
SVDquartets tree of the *de novo* alignment produced by the UNEAK pipeline for *Molossus*. Numbers represent bootstrap support values.

**Fig. 3:**
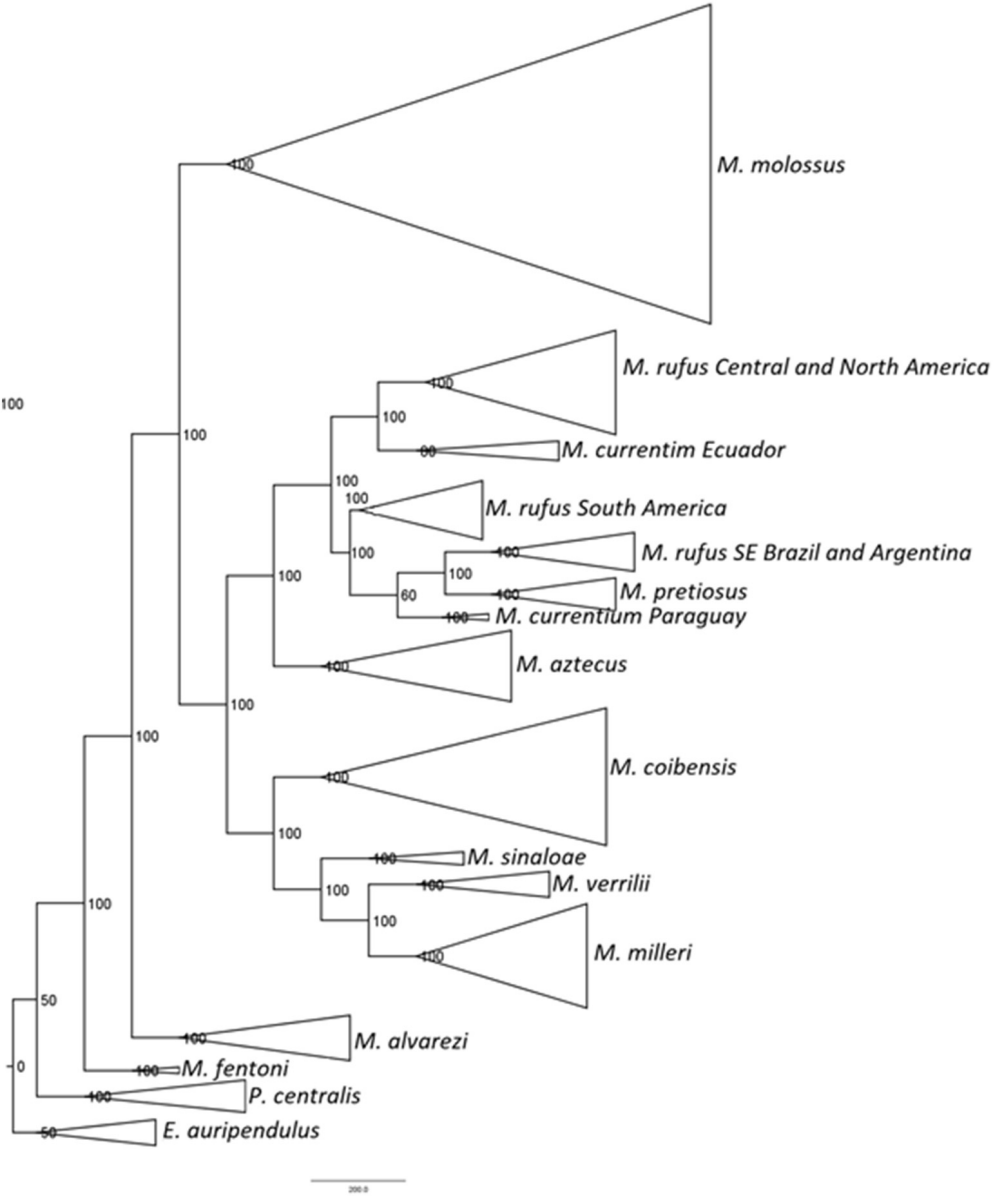
SVDquartets tree with the alignment using the *Myotis* reference genome produced by the Discovery pipeline for *Molossus*. Numbers represent bootstrap support values.

**Fig. 4:**
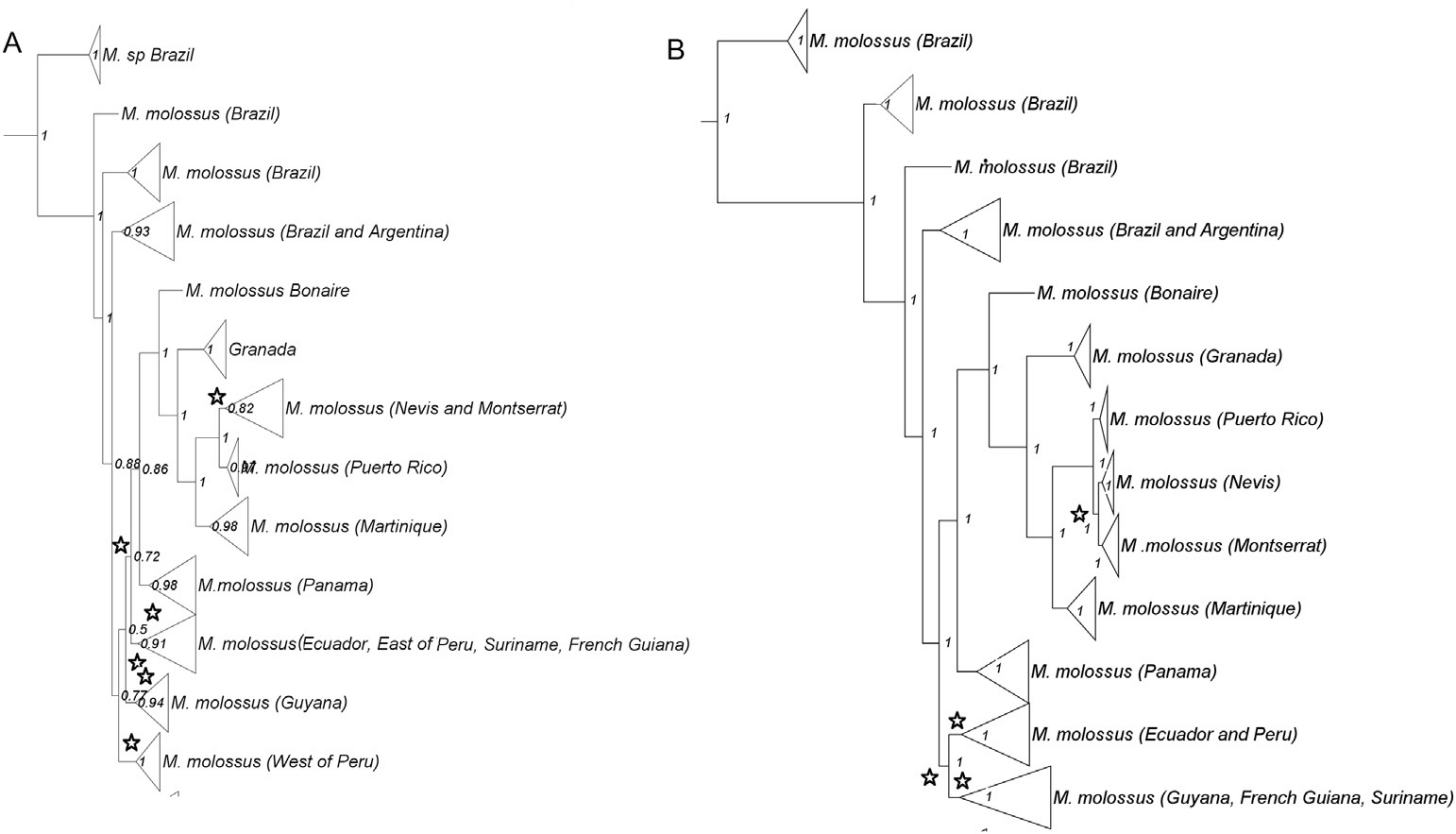
Internal nodes of the *M. molossus* clade produced by the Maximum likelihood and SVDquartets trees. A- De novo alignment; B- Alignment using the reference genome. Numbers represent bootstrap support values and  represents nodes with divergence between the two approaches.

**Fig. 5:**
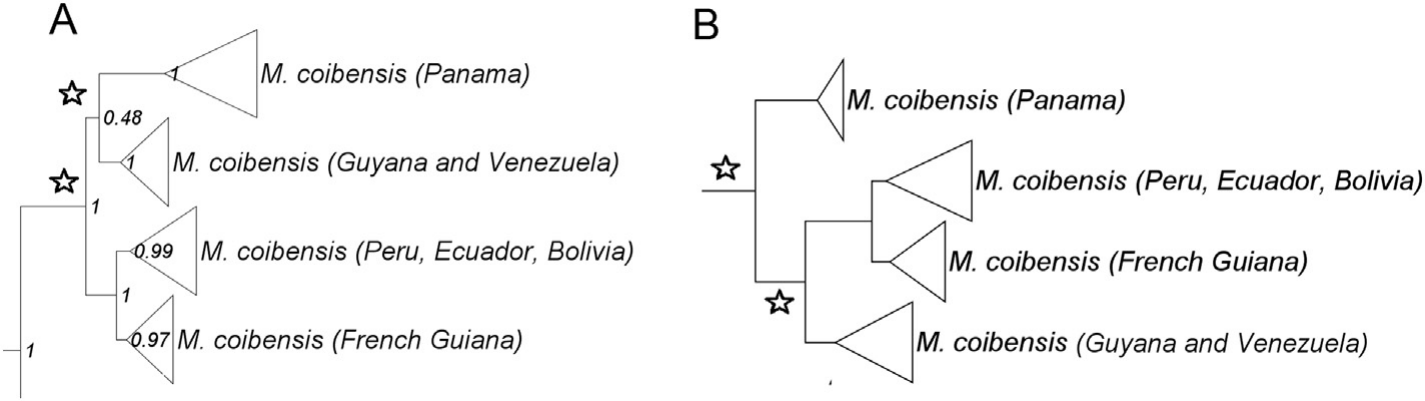
Internal nodes of the *M. coibensis* clade produced by the Maximum likelihood and SVDquartets trees. A- De novo alignment; B- Alignment using the reference genome. Numbers represent bootstrap support values and  represents nodes where the two approaches differ.

*Molossus* is a common and diverse genus of bat in the family Molossidae. Due to the low genetic variation among some species, traditional Sanger methods could not resolve the evolutionary relationships within the genus [6,7]. Herein we estimated the relationships within *Molossus* based on 189 specimens, including outgroups (*Promops* and *Eumops*), distributed in North, Central, and South America, and Caribbean islands. We present SVDquartets and Maximum Likelihood phylogenetic trees for *Molossus* based on genotype by sequencing approach assembled *de novo* and with a reference genome (*Myotis brandtii*) (Fig. 1, Fig. 2, Fig. 3). Phylogenetic relationships using the *de novo* alignment and the Maximum Likelihood approach and co-phylogenetic plots showing difference in structure between alignments are available in Loureiro et al. [1]. All phylogentic trees show well supported species boundaries and relationships among species. We also show relationships within *M. molossus* and *M. coibensis* produced by the Maximum Likelihood trees, highlighting divergent relationships recovered between approaches (Fig. 4, Fig. 5). The difference within internal clades in the SVDquartets approaches were identical to the maximum likelihood approach and are not shown. In addition, we present the specimen vouchers used in the genetic analyses (Supplementary material 1), the species identification, the country and the coordinates where the specimens were collected. Specimens used in the morphological analyses used to confirm the identification of the clades recovered in the phylogenies are presented in Supplementary material 2.

## 2 Experimental design, materials, and methods

Tissue samples were obtained from 189 specimens of *Molossus* (Supplementary material 1) [1]. Individuals from two other species of molossids bats, *Promops centralis* and *Eumops auripendulus*, were also included, and used as outgroups [8,9]. We isolated the DNA with the Qiagen DNeasy extraction kit (Qiagen, Inc. Valencia, CA, USA) following the manufacturer's instructions.

A Nanodrop spectrophotometer (Nanodrop Technologies) was used to quantify the total DNA and the quality of the DNA was checked manually on agarose gels. For library preparation, we used thirty microlitres of high quality (>100 ng/ul) DNA per individual. Libraries preparation through the genotyping by sequencing approach (GBS) were conducted in the Cornell Institute of Genomic Diversity (IGD) on an Illumina HiSeq 2000 following the protocol described by Elshire et al. [2].

The raw sequence files produced by Illumina were sequenced using two approaches. First, we aligned the data using a reference genome (*Myotis brandtii*) in the Discovery pipeline [10], available as part of the TASSEL 3.0 software [3]. As a second approach we also aligned the tags *de novo* using the Universal Network-Enabled Analysis Kit (UNEAK) pipelines also on TASSEL [3]. To remove sequencing errors, we filtered the data following Loureiro et al. [11]. We removed SNPs with heterozygosity >0.01 and minor allele frequency (MAF) > 0.02. SNPs with more than 50% of missing data were also removed. We set as missing data alleles with depth coverage lower than six for the Discovery and lower than seven for the UNEAK pipeline. The final filtered data recovered 71,801 SNPs with UNEAK pipeline and 27,323 SNPs with the Discovery pipeline. To decrease linkage disequilibrium, alleles that were less than 128 bp apart were discarded. The final genomic dataset for the UNEAK pipeline yielded 29,448 variants SNPs, and for the Discovery pipeline yielded variants 15,569 SNPs. The variant call format (VCF) file containing the variants might be opened using the TASSEL software [3], as used in this study, or any package designed for working with VCF files, such as VCFtools [12].

Evolutionary relationships among species of *Molossus* were investigated though a coalescent approach, which considers differences in genealogical histories based on individual loci. This analysis was conducted using SVDquartets [5] implemented in PAUP 4.0 [13]. To access topological convergence, four independent runs were conducted, each including 500 bootstrap replicates and exhaustive quartet sampling. Phylogenetic relationships within *Molossus* were also recovered using the Maximum Likelihood approach (ML) implemented in FastTree [4]. We estimated the model of nucleotide evolution (GTR + gamma) using Partition Finder 1.0.1 [14]. Trees were visualised using FigTree v. 1.4.3.
