# Supplementary material for: Next generation sequencing data in the phylogenetic relationships of the genus Molossus (Chiroptera, Molossidae)
Source: Data Brief. 2020 Feb 14;29:105276. doi: 10.1016/j.dib.2020.105276 (PMC7038581; doi:10.1016/j.dib.2020.105276)
Supplement: Multimedia component 1 [file mmc1.docx]

Supplementary material 1 - Specimen vouchers, species identification, and country of origin for *Molossus* used in the genetic analyses.

| **Sample Name** | **Country** | **Genus** | **Species** | **Locality** | **Latitude** | **Longitude** |
| --- | --- | --- | --- | --- | --- | --- |
| ROM 103342 | Guyana | *Eumops* | *auripendulus* | Surama | 4.133 | -59.033 |
| ROM 108464 | Guyana | *Eumops* | *auripendulus* | Iwokrama Reserve | 4.366 | -58.75 |
| AMNH 269107 | French Guiana | *Molossus* | *alvarezi* | Rio Lagartos | 21.596 | -88.158 |
| AMNH 267549 | French Guiana | *Molossus* | *alvarezi* | Rio Lagartos | 21.596 | -88.158 |
| AMNH 269110 | French Guiana | *Molossus* | *alvarezi* | Rio Lagartos | 21.596 | -88.158 |
| ROM 126064 | Mexico | *Molossus* | *alvarezi* | Rio Lagartos | 21.596 | -88.158 |
| ROM126070 | Mexico | *Molossus* | *alvarezi* | Rio Lagartos | 21.596 | -88.158 |
| ROM126071 | Mexico | *Molossus* | *alvarezi* | Rio Lagartos | 21.596 | -88.158 |
| ROM 126072 | Mexico | *Molossus* | *alvarezi* | Rio Lagartos | 21.596 | -88.158 |
| ROM 126083 | Mexico | *Molossus* | *alvarezi* | Rio Lagartos | 21.596 | -88.158 |
| ROM 126084 | Mexico | *Molossus* | *alvarezi* | Rio Lagartos | 21.596 | -88.158 |
| UFLA 399 | Brazil | *Molossus* | *aztecus* | Lavras | -21.245 | -44.999 |
| UFLA 536 | Brazil | *Molossus* | *aztecus* | Lavras | -21.245 | -44.999 |
| UFLA 693 | Brazil | *Molossus* | *aztecus* | Lavras | -21.245 | -44.999 |
| UFLA 988 | Brazil | *Molossus* | *aztecus* | Lavras | -21.245 | -44.999 |
| UFLA 1145 | Brazil | *Molossus* | *aztecus* | Merlieria | -19.171 | -42.75 |
| UFLA 1144 | Brazil | *Molossus* | *aztecus* | Merlieria | -19.171 | -42.75 |
| UFLA 1146 | Brazil | *Molossus* | *aztecus* | Merlieria | -19.171 | -42.75 |
| UFLA 1797 | Brazil | *Molossus* | *aztecus* | Merlieria | -19.171 | -42.75 |
| UFLA_1826 | Brazil | *Molossus* | *aztecus* | Merlieria | -19.171 | -42.75 |
| UFLA 415 | Brazil | *Molossus* | *aztecus* | Lavras | -21.245 | -44.999 |
| UFLA 416 | Brazil | *Molossus* | *aztecus* | Lavras | -21.245 | -44.999 |
| TTU 33536 | Mexico | *Molossus* | *aztecus* | Tehuantepec | 16.333 | -95.233 |
| TTU 33530 | Mexico | *Molossus* | *aztecus* | Tehuantepec | 16.333 | -95.233 |
| CRD 2494 | Mexico | *Molossus* | *aztecus* | Durango | 24.033 | -104.667 |
| TTU 33531 | Mexico | *Molossus* | *aztecus* | Tehuantepec | 16.333 | -95.233 |
| ROM 105638 | Ecuador | *Molossus* | *coibensis* | Napo | 39.000 | -76.270 |
| ROM 105637 | Ecuador | *Molossus* | *coibensis* | Napo | 39.000 | -76.270 |
| ROM 105688 | Ecuador | *Molossus* | *coibensis* | Napo | 3.900 | -76.270 |
| MHNG19884.011 | French Guiana | *Molossus* | *coibensis* | Cacao | 4.568 | -52.468 |
| MNHG19883.014 | French Guiana | *Molossus* | *coibensis* | Cacao | 4.568 | -52.468 |
| MHNG1984.007 | French Guiana | *Molossus* | *coibensis* | Kaw | 4.550 | -52.167 |
| AMNH267262 | French Guiana | *Molossus* | *coibensis* | Cayenne | 3.933 | -53.008 |
| AMNH 269105 | French Guiana | *Molossus* | *coibensis* | Cayenne | 3.933 | -53.008 |
| ROM 119012 | Guyana | *Molossus* | *coibensis* | Dadanawa Ranch | 2.493 | -59.313 |
| ROM 53797 | Peru | *Molossus* | *coibensis* | Loreto | 4.899 | -73.650 |
| PAN 88067 | Panama | *Molossus* | *coibensis* | Gamboa | 9.116 | -79.699 |
| PAN B3910 | Panama | *Molossus* | *coibensis* | Gamboa | 9.116 | -79.699 |
| EBD 21603 | Panama | *Molossus* | *coibensis* | Gamboa | 9.116 | -79.699 |
| ROM 122087 | Peru | *Molossus* | *coibensis* | Loreto | 4.899 | -73.650 |
| ROM 122091 | Peru | *Molossus* | *coibensis* | Loreto | 4.899 | -73.650 |
| ROM 122177 | Peru | *Molossus* | *coibensis* | Loreto | 4.899 | -73.650 |
| TTU 134664 | Ecuador | *Molossus* | *bondae* | Guayas | -2.600 | -79.900 |
| TTU 134663 | Ecuador | *Molossus* | *bondae* | Guayas | -2.600 | -79.900 |
| TTU 134665 | Ecuador | *Molossus* | *bondae* | Guayas | -2.600 | -79.900 |
| TTU 12386 | Nicaragua | *Molossus* | *bondae* | Zelaya | 12.136 | -86.251 |
| TTU 61008 | Paraguay | *Molossus* | *currentium* | Alto Paraguay | -21.733 | -57.917 |
| TK 61016 | Paraguay | *Molossus* | *currentium* | Alto Paraguay | -21.733 | -57.917 |
| TK 61025 | Paraguay | *Molossus* | *currentium* | Alto Paraguay | -21.733 | -57.917 |
| ROM 118821 | Ecuador | *Molossus* | *fentoni* | Orellana | 0.53 | -75.97 |
| ROM 109176 | Guyana | *Molossus* | *fentoni* | Potario-Siparuni | 4.7856 | -59.288 |
| ROM 122583 | Guyana | *Molossus* | *fentoni* | Potario-Siparuni | 4.7856 | -59.288 |
| ROM 125985 | Cayman Islands | *Molossus* | *milleri* | Cayman Brac | 19.699 | -79.865 |
| ROM 125983 | Cayman Islands | *Molossus* | *milleri* | Cayman Brac | 19.699 | -79.865 |
| ROM 125955 | Cayman Islands | *Molossus* | *milleri* | Grand Cayman | 19.31433 | -81.169 |
| ROM 125963 | Cayman Islands | *Molossus* | *milleri* | Grand Cayman | 19.332 | -81.104 |
| 125957 ROM | Cayman Islands | *Molossus* | *milleri* | Grand Cayman | 19.314 | -81.169 |
| ROM 125959 | Cayman Islands | *Molossus* | *milleri* | Grand Cayman | 19.271 | -81.281 |
| ROM 125984 | Cayman Islands | *Molossus* | *milleri* | Cayman Brac | 19.699 | -79.856 |
| ROM 125964 | Cayman Islands | *Molossus* | *milleri* | Grand Cayman | 19.332 | -81.104 |
| SMNH 59028 | Cuba | *Molossus* | *milleri* | Guantanamo Bay | 19.900 | -75.150 |
| SMNH 519030 | Cuba | *Molossus* | *milleri* | Guantanamo Bay | 19.900 | -75.150 |
| SMNH 599033 | Cuba | *Molossus* | *milleri* | Guantanamo Bay | 19.900 | -75.150 |
| LMNH 54981 | Cuba | *Molossus* | *milleri* | Guantanamo Bay | 19.900 | -75.150 |
| TTU 52647 | Cuba | *Molossus* | *milleri* | Guantanamo Bay | 19.900 | -75.150 |
| TTU 32081 | Cuba | *Molossus* | *milleri* | Guantanamo Bay | 19.900 | -75.150 |
| TTU 68821 | Cuba | *Molossus* | *milleri* | Guantanamo Bay | 19.900 | -75.150 |
| TTU 22195 | Jamaica | *Molossus* | *milleri* | St. Ann | 18.436 | -77.201 |
| ROM 120849 | Jamaica | *Molossus* | *milleri* | Portland | 18.14347 | -76.373 |
| ROM 120796 | Jamaica | *Molossus* | *milleri* | Saint Elizabeth | 18.227 | 77.75446 |
| ROM 120821 | Jamaica | *Molossus* | *milleri* |  |  |  |
| ROM 120850 | Jamaica | *Molossus* | *milleri* | Portland | 18.14347 | -76.373 |
| ROM 120798 | Jamaica | *Molossus* | *milleri* | Portland | 18.14347 | -76.373 |
| OKLA 7438 | Argentina | *Molossus* | *molossus* | Jujuy | -32.6 | -63.883 |
| TTU 151411* | Barbados | *Molossus* | *molossus* | St. Thomas Parish |  |  |
| USNM 584499 | Bolivia | *Molossus* | *molossus* | Santa Cruz | -17.786 | -63.181 |
| MSB 210952 | Bolivia | *Molossus* | *molossus* | Santa Cruz | -17.786 | -63.181 |
| ROM 125468 | Bonaire | *Molossus* | *molossus* | 0 | 12.202 | -68.262 |
| MZV 1853731 | Brazil | *Molossus* | *molossus* | Pernambuco | -8.472 | -37.947 |
| UFLA 1781 | Brazil | *Molossus* | *molossus* | Minas Gerais | -19.717 | -42.75 |
| CRB 2030 | Brazil | *Molossus* | *molossus* | Not provided | Not provided | Not provided |
| UFLA 1929 | Brazil | *Molossus* | *molossus* | Minas Gerais | -19.717 | -42.75 |
| UFMG 5275 | Brazil | *Molossus* | *molossus* | Minas Gerais | -18.919 | -48.277 |
| ROM 104022 | Ecuador | *Molossus* | *molossus* | Napo | -3.99 | -76.27 |
| FMNH 213848 | Ecuador | *Molossus* | *molossus* | Tiguino | -0.462 | -76.993 |
| USNM 574566 | Ecuador | *Molossus* | *molossus* | Orellana | -0.466 | -76.987 |
| AMNH 269102 | French Guiana | *Molossus* | *molossus* | Cayenne | -3.933 | -53.088 |
| MHNG 1885.023 | French Guiana | *Molossus* | *molossus* | Awala-Yalimapo | -5.7411 | -53.928 |
| MHNH 2004-356 | French Guiana | *Molossus* | *molossus* | Angoulème | -5.4 | -53.65 |
| MHNG 1972-021 | French Guiana | *Molossus* | *molossus* | Cacao | -4.568 | -52.468 |
| TTU 18551 | Grenada | *Molossus* | *molossus* | St George | 12.056 | -61.748 |
| TTU 18553 | Grenada | *Molossus* | *molossus* | St George | 12.056 | -61.748 |
| TTU 18556 | Grenada | *Molossus* | *molossus* | St George | 12.056 | -61.748 |
| TTU 18557 | Grenada | *Molossus* | *molossus* | St George | 12.056 | -61.748 |
| ROM 97775 | Guyana | *Molossus* | *molossus* | Upper Takutu-Upper Essequibo | -3.23 | -59.48 |
| ROM 98716 | Guyana | *Molossus* | *molossus* | Barima-Waini | -7.34 | -59.09 |
| ROM 122608 | Guyana | *Molossus* | *molossus* | Upper Takutu-Upper Essequibo | -2.182 | -59.337 |
| ROM 107256 | Guyana | *Molossus* | *molossus* | Potaro-Siparuni | -4.4 | -58.41 |
| ROM 103556 | Guyana | *Molossus* | *molossus* | Upper Demerara-Berbice | -5.18 | -58.42 |
| ROM 98703 | Guyana | *Molossus* | *molossus* | Barima-Waini | -7.34 | -59.09 |
| ROM 108127 | Guyana | *Molossus* | *molossus* | Cuyuni-Mazaruni | -5.52 | -60.37 |
| MHNH 2005-791 | Martinique | *Molossus* | *molossus* | Le Precheur | 14.8 | -61.217 |
| MHNH 2055-793 | Martinique | *Molossus* | *molossus* | Le Precheur | 14.8 | -61.217 |
| MHNH V-2091 | Martinique | *Molossus* | *molossus* | Morne Rouge | 14.767 | -61.133 |
| MNHN V-3486 | Martinique | *Molossus* | *molossus* | Morne Rouge | 14.767 | -61.133 |
| ECO-SC-M 3111 | Mexico | *Molossus* | *molossus* | Tabasco | 17.8 | -91.533 |
| ECO-SC-M 3115 | Mexico | *Molossus* | *molossus* | Tabasco | 17.8 | -91.533 |
| ECO-SC-M 5675* | Mexico | *Molossus* | *molossus* | Quintana Roo | 19.578 | -88.045 |
| MZV 166187 | Montserrat | *Molossus* | *molossus* | Belham River | 16.749 | -62.193 |
| MZV 166225 | Montserrat | *Molossus* | *molossus* | Belham River | 16.749 | -62.193 |
| TTU 129173 | Montserrat | *Molossus* | *molossus* | Belham River | 16.749 | -62.193 |
| TTU 151275* | Montserrat | *Molossus* | *molossus* | Castries | 13.996 | -61.006 |
| ROM 125988 | Nevis | *Molossus* | *molossus* | Newcastle | 17.191 | -62.586 |
| ROM 125990 | Nevis | *Molossus* | *molossus* | Newcastle | 17.196 | -62.596 |
| ROM 125995 | Nevis | *Molossus* | *molossus* | Newcastle | 17.196 | -62.596 |
| ROM 126004 | Nevis | *Molossus* | *molossus* | Pond Hill | 17.124 | -62.593 |
| TTU 29603 | Nicaragua | *Molossus* | *molossus* | Rivas | 11.47 | -86.125 |
| ROM 104194 | Panama | *Molossus* | *molossus* | Gamboa | 9.06 | -79.42 |
| TTU 12427 | Panama | *Molossus* | *molossus* | Chiriqui | 8.517 | -82.617 |
| TTU 12428 | Panama | *Molossus* | *molossus* | Chiriqui | 8.517 | -82.617 |
| TTU 62822 | Paraguay | *Molossus* | *molossus* | Boqueron | -22.452 | -62.348 |
| TTU 129172* | Paraguay | *Molossus* | *molossus* | Boqueron | -22.452 | -62.348 |
| ROM 122253 | Peru | *Molossus* | *molossus* | Loreto | -9.899 | -73.65 |
| MZV 163935 | Peru | *Molossus* | *molossus* | Loreto | -9.899 | -73.65 |
| ROM 122256* | Peru | *Molossus* | *molossus* | Loreto | -9.899 | -73.65 |
| MZV 168936 | Peru | *Molossus* | *molossus* | Lambayerque | -6.7 | -79.9 |
| LMNH 7545* | Peru | *Molossus* | *molossus* | Amazonas | -9.825 | -77.948 |
| USNM 581971 | Peru | *Molossus* | *molossus* | Cordillera Del Condor | -4.095 | -78.393 |
| ROM 122131 | Peru | *Molossus* | *molossus* | Loreto | -9.899 | -73.65 |
| FMNH 206534 | Puerto Rico | *Molossus* | *molossus* | Vieques Island | 18.125 | -65.442 |
| FMNH 206535 | Puerto Rico | *Molossus* | *molossus* | Vieques Island | 18.125 | -65.442 |
| TTU 151296* | St Lucia | *Molossus* | *molossus* | Castries | 13.996 | -61.006 |
| ROM 120414 | Suriname | *Molossus* | *molossus* | Sipaliwini | -2.026 | -56.124 |
| ROM 120413 | Suriname | *Molossus* | *molossus* | Sipaliwini | -2.026 | -56.124 |
| UFMG 5268 | Brazil | *Molossus* | *pretiosus* | Juiz de Fora | -21.749 | -43.349 |
| UFMG 5177 | Brazil | *Molossus* | *pretiosus* | Fortaleza de Minas | -20.2 | -41.667 |
| UFMG 5207 | Brazil | *Molossus* | *pretiosus* | Uberlandia | -18.913 | -48.266 |
| UFMG 5266 | Brazil | *Molossus* | *pretiosus* | Uberlandia | -18.913 | -48.266 |
| MCNas_209 | Brazil | *Molossus* | *pretiosus* | Jaiba | -12.2 | -38.883 |
| UFMG 5269 | Brazil | *Molossus* | *pretiosus* | Uberlandia | -18.913 | -48.266 |
| TTU 12555 | Costa Rica | *Molossus* | *pretiosus* | Guanacaste | 10.63 | -85.439 |
| TTU 12565 | Costa Rica | *Molossus* | *pretiosus* | Guanacaste | 10.63 | -85.439 |
| TTU 12343 | Nicaragua | *Molossus* | *pretiosus* | Boaco | 12.472 | -85.659 |
| TTU 29780 | Nicaragua | *Molossus* | *pretiosus* | Boaco | 12.472 | -85.659 |
| TTU 12342 | Nicaragua | *Molossus* | *pretiosus* | Boaco | 12.472 | -85.659 |
| OKLA 1617 | Argentina | *Molossus* | *rufus* | Chaco | -27.46 | -58.983 |
| UFLA 1815 | Brazil | *Molossus* | *rufus* | Merlieria | -19.171 | -42.75 |
| UFLA 1926 | Brazil | *Molossus* | *rufus* | Merlieria | -19.171 | -42.75 |
| UFLA 1927 | Brazil | *Molossus* | *rufus* | Merlieria | -19.171 | -42.75 |
| UFMG 5272 | Brazil | *Molossus* | *rufus* | Governador Valadares | -18.851 | -41.949 |
| MZV 185589 | Brazil | *Molossus* | *rufus* | Barra | -12.7 | -41.55 |
| UFMG 5273 | Brazil | *Molossus* | *rufus* | Uberlandia | -18.913 | -48.266 |
| ROM 101279 | El Salvador | *Molossus* | *rufus* | El Imposible, El Refugio | 13.84 | -89.95 |
| ROM 101280 | El Salvador | *Molossus* | *rufus* | El Imposible, El Refugio | 13.84 | -89.95 |
| AMNH 268600 | French Guiana | *Molossus* | *rufus* | Paracou | 5.383 | -52.9 |
| MNHG 1990-009 | French Guiana | *Molossus* | *rufus* | Cayenne | 3.933 | -53.008 |
| ECO 1634 | Guatemala | *Molossus* | *rufus* | San Francisco del Mar | 15.833 | -88.417 |
| ROM F39068 | Guyana | *Molossus* | *rufus* | Iwokrama Reserve | 4.366 | -58.75 |
| ROM 108419 | Guyana | *Molossus* | *rufus* | Iwokrama Reserve | 4.366 | -58.75 |
| ECO-SC-M 1635 | Mexico | *Molossus* | *rufus* | San Francisco del Mar | 15.833 | -88.417 |
| ECO-SC-M 1637 | Mexico | *Molossus* | *rufus* | San Francisco del Mar | 15.833 | -88.417 |
| CRD 5093 | Mexico | *Molossus* | *rufus* | Camotete | 26 | -97.8 |
| ECO-SC-M 5873 | Mexico | *Molossus* | *rufus* | Othón P. Blanco | 18.514 | -88.304 |
| ECO-SC-M 6586 | Mexico | *Molossus* | *rufus* | Champotón | 19.35 | -90.717 |
| ROM 96186 | Mexico | *Molossus* | *rufus* | Lazaro Cardenas | 17.97 | -102.221 |
| ROM 126060 | Mexico | *Molossus* | *rufus* | Playa del Carmen | 20.627 | -87.079 |
| MZV 223392 | Guatemala | *Molossus* | *rufus* | Turicentro Guaytán | 14.94 | -89.969 |
| ECO-SC-M 3092 | Mexico | *Molossus* | *rufus* | Balancan | 17.799 | -91.533 |
| FMNH 174943 | Peru | *Molossus* | *rufus* | Cusco | -13.521 | -71.969 |
| FMNH 174944 | Peru | *Molossus* | *rufus* | Cusco | -13.521 | -71.969 |
| ROM 122090 | Peru | *Molossus* | *rufus* | Loreto | 4.899 | -73.650 |
| ROM 105682 | Suriname | *Molossus* | *rufus* | Napo | 39.000 | -76.270 |
| TTU 19501 | Venezuela | *Molossus* | *rufus* | Barinas | 8.622 | -70.207 |
| TTU 19502 | Venezuela | *Molossus* | *rufus* | Barinas | 8.622 | -70.207 |
| MZFC 6475 | Mexico | *Molossus* | *sinaloae* | Tecpan de Galeana | 17.25 | -100.683 |
| MZFC 3909 | Mexico | *Molossus* | *sinaloae* | Arteaga | 25.445 | -100.847 |
| ECO-SC-M1413 | Mexico | *Molossus* | *sinaloae* | Pijijiapan | 15.7 | -93.233 |
| ROM 107901 | Venezuela | *Molossus* | *sp* | Amazonas | 6.03 | -67.25 |
| ROM 107900 | Venezuela | *Molossus* | *sp* | Amazonas | 6.03 | -67.25 |
| ROM 107869 | Venezuela | *Molossus* | *sp* | Amazonas | 6.03 | -67.25 |
| ROM 125287 | Dominican Republic | *Molossus* | *verrilli* | Santo Domingo | 18.483 | -69.929 |
| ROM 125289 | Dominican Republic | *Molossus* | *verrilli* | Santo Domingo | 18.483 | -69.929 |
| ROM 125385 | Dominican Republic | *Molossus* | *verrilli* | Puerto Plata | 19.781 | -70.687 |
| ROM 125387 | Dominican Republic | *Molossus* | *verrilli* | Puerto Plata | 19.781 | -70.687 |
| ROM 125286 | Dominican Republic | *Molossus* | *verrilli* | Santo Domingo | 18.483 | -69.929 |
| ROM 106035 | Ecuador | *Promops* | *centralis* | Napo | 39.000 | -76.270 |
| ROM 118824 | Ecuador | *Promops* | *centralis* | Orellana | 0.53 | -75.97 |
