# Supplementary material for: Next generation sequencing data in the phylogenetic relationships of the genus Molossus (Chiroptera, Molossidae)
Source: Data Brief. 2020 Feb 14;29:105276. doi: 10.1016/j.dib.2020.105276 (PMC7038581; doi:10.1016/j.dib.2020.105276)
Supplement: Multimedia component 2 [file mmc2.docx]

Supplementary material 2 - Specimen vouchers, species identification, and country of origin for *Molossus* used in the morphological analysis.

| **Institution** | **Catalog Number** | **Country** | **Genus** | **Species** |
| --- | --- | --- | --- | --- |
| AMNH | M-146587 | Belize | *Molossus* | *alvarezi* |
| AMNH | M-146588 | Belize | *Molossus* | *alvarezi* |
| AMNH | M-267547 | French Guiana | *Molossus* | *alvarezi* |
| AMNH | M-269110 | French Guiana | *Molossus* | *alvarezi* |
| AMNH | M-267549 | French Guiana | *Molossus* | *alvarezi* |
| SMNH | 260048 | Guatemala | *Molossus* | *alvarezi* |
| SMNH | 260047 | Guatemala | *Molossus* | *alvarezi* |
| AMNH | M-265132 | Honduras | *Molossus* | *alvarezi* |
| AMNH | M-265133 | Honduras | *Molossus* | *alvarezi* |
| SMNH | 461105 | Honduras | *Molossus* | *alvarezi* |
| SMNH | 148773 | Honduras | *Molossus* | *alvarezi* |
| ROM | 126070 | Mexico | *Molossus* | *alvarezi* |
| ROM | 126071 | Mexico | *Molossus* | *alvarezi* |
| ROM | 126072 | Mexico | *Molossus* | *alvarezi* |
| ROM | 126083 | Mexico | *Molossus* | *alvarezi* |
| ROM | 126084 | Mexico | *Molossus* | *alvarezi* |
| AMNH | M-204985 | Mexico | *Molossus* | *alvarezi* |
| AMNH | M-204986 | Mexico | *Molossus* | *alvarezi* |
| AMNH | M-41190 | Nicaragua | *Molossus* | *alvarezi* |
| AMNH | M-41191 | Nicaragua | *Molossus* | *alvarezi* |
| AMNH | M-41194 | Nicaragua | *Molossus* | *alvarezi* |
| AMNH | M-41195 | Nicaragua | *Molossus* | *alvarezi* |
| AMNH | M-179987 | Trinidad and Tobago | *Molossus* | *alvarezi* |
| AMNH | M-256340 | Trinidad and Tobago | *Molossus* | *alvarezi* |
| UFMS | MA1538 | Brazil | *Molossus* | *aztecus* |
| MZUSP | 8329 | Brazil | *Molossus* | *aztecus* |
| MZUSP | 10269 | Brazil | *Molossus* | *aztecus* |
| MZUSP | 8346 | Brazil | *Molossus* | *aztecus* |
| MZUSP | 10270 | Brazil | *Molossus* | *aztecus* |
| UNESP | 12788 | Brazil | *Molossus* | *aztecus* |
| UNESP | 12781 | Brazil | *Molossus* | *aztecus* |
| UNESP | 13176 | Brazil | *Molossus* | *aztecus* |
| UNESP | 13271 | Brazil | *Molossus* | *aztecus* |
| MNRIO | 23063 | Brazil | *Molossus* | *aztecus* |
| CMUFLA | 1051 | Brazil | *Molossus* | *aztecus* |
| UFMS | MA635 | Brazil | *Molossus* | *aztecus* |
| UFMS | MA1545 | Brazil | *Molossus* | *aztecus* |
| UFMS | MA1539 | Brazil | *Molossus* | *aztecus* |
| MZUSP | 8330 | Brazil | *Molossus* | *aztecus* |
| UFMS | CHR35 | Brazil | *Molossus* | *aztecus* |
| MZUSP | 16834 | Brazil | *Molossus* | *aztecus* |
| MZUSP | 16861 | Brazil | *Molossus* | *aztecus* |
| MZUSP | 16841 | Brazil | *Molossus* | *aztecus* |
| MZUSP | 16800 | Brazil | *Molossus* | *aztecus* |
| MZUSP | 16814 | Brazil | *Molossus* | *aztecus* |
| MZUSP | 14897 | Brazil | *Molossus* | *aztecus* |
| MZUSP | 14896 | Brazil | *Molossus* | *aztecus* |
| MZUSP | 14904 | Brazil | *Molossus* | *aztecus* |
| MZUSP | 1487 | Brazil | *Molossus* | *aztecus* |
| CMUFLA | 399 | Brazil | *Molossus* | *aztecus* |
| UFRRJ | 6294 | Brazil | *Molossus* | *aztecus* |
| UFRRJ | 6295 | Brazil | *Molossus* | *aztecus* |
| UFRRJ | 6298 | Brazil | *Molossus* | *aztecus* |
| UFRRJ | 425 | Brazil | *Molossus* | *aztecus* |
| UFRRJ | 424 | Brazil | *Molossus* | *aztecus* |
| UFRRJ | 646 | Brazil | *Molossus* | *aztecus* |
| UFRRJ | 420 | Brazil | *Molossus* | *aztecus* |
| CMUFLA | 400 | Brazil | *Molossus* | *aztecus* |
| UFMS | MA697 | Brazil | *Molossus* | *aztecus* |
| MZUSP | 1309 | Brazil | *Molossus* | *aztecus* |
| CMUFLA | RG13269 | Brazil | *Molossus* | *aztecus* |
| CMUFLA | RG13272 | Brazil | *Molossus* | *aztecus* |
| CMUFLA | RG13274 | Brazil | *Molossus* | *aztecus* |
| CMUFLA | RG13264 | Brazil | *Molossus* | *aztecus* |
| CMUFLA | RG13276 | Brazil | *Molossus* | *aztecus* |
| CMUFLA | RG13173 | Brazil | *Molossus* | *aztecus* |
| CMUFLA | RG13180 | Brazil | *Molossus* | *aztecus* |
| MZUSP | 1309 | Brazil | *Molossus* | *aztecus* |
| SMNH | 391164 | Brazil | *Molossus* | *aztecus* |
| SMNH | 391163 | Brazil | *Molossus* | *aztecus* |
| SMNH | 301162 | Brazil | *Molossus* | *aztecus* |
| SMNH | 314301 | Brazil | *Molossus* | *aztecus* |
| SMNH | 391175 | Brazil | *Molossus* | *aztecus* |
| SMNH | 391167 | Brazil | *Molossus* | *aztecus* |
| SMNH | 391171 | Brazil | *Molossus* | *aztecus* |
| SMNH | 39174 | Brazil | *Molossus* | *aztecus* |
| AMNH | 172045 | Mexico | *Molossus* | *aztecus* |
| AMNH | 172044 | Mexico | *Molossus* | *aztecus* |
| AMNH | 206873 | Mexico | *Molossus* | *aztecus* |
| AMNH | 206876 | Mexico | *Molossus* | *aztecus* |
| AMNH | 171597 | Mexico | *Molossus* | *aztecus* |
| AMNH | 171605 | Mexico | *Molossus* | *aztecus* |
| AMNH | 171639 | Mexico | *Molossus* | *aztecus* |
| AMNH | 171640 | Mexico | *Molossus* | *aztecus* |
| AMNH | M-27332 | Mexico | *Molossus* | *aztecus* |
| AMNH | M-171597 | Mexico | *Molossus* | *aztecus* |
| AMNH | M-171605 | Mexico | *Molossus* | *aztecus* |
| AMNH | M-175274 | Mexico | *Molossus* | *aztecus* |
| AMNH | M-190188 | Mexico | *Molossus* | *aztecus* |
| MZUSP | 2672 | Brazil | *Molossus* | *coibensis* |
| UFMG | 3411 | Brazil | *Molossus* | *coibensis* |
| UFMG | 3404 | Brazil | *Molossus* | *coibensis* |
| UFMG | 3405 | Brazil | *Molossus* | *coibensis* |
| MZUSP | 28782 | Brazil | *Molossus* | *coibensis* |
| MZUSP | 28689 | Brazil | *Molossus* | *coibensis* |
| UFMG | 3350 | Brazil | *Molossus* | *coibensis* |
| UFMS | MA1154 | Brazil | *Molossus* | *coibensis* |
| AMNH | 269105 | French Guiana | *Molossus* | *coibensis* |
| AMNH | M-269105 | French Guiana | *Molossus* | *coibensis* |
| AMNH | 217449 | Guatemala | *Molossus* | *coibensis* |
| AMNH | 217448 | Guatemala | *Molossus* | *coibensis* |
| AMNH | 217446 | Guatemala | *Molossus* | *coibensis* |
| AMNH | 217445 | Guatemala | *Molossus* | *coibensis* |
| AMNH | 217444 | Guatemala | *Molossus* | *coibensis* |
| AMNH | 217443 | Guatemala | *Molossus* | *coibensis* |
| AMNH | M-217450 | Guatemala | *Molossus* | *coibensis* |
| AMNH | M-217451 | Guatemala | *Molossus* | *coibensis* |
| AMNH | M-217452 | Guatemala | *Molossus* | *coibensis* |
| AMNH | M-217453 | Guatemala | *Molossus* | *coibensis* |
| AMNH | 18732 | Panama | *Molossus* | *coibensis* |
| AMNH | 18733 | Panama | *Molossus* | *coibensis* |
| AMNH | 173919 | Panama | *Molossus* | *coibensis* |
| AMNH | 183864 | Panama | *Molossus* | *coibensis* |
| AMNH | 183867 | Panama | *Molossus* | *coibensis* |
| SMNH | 314299 | Panama | *Molossus* | *coibensis* |
| SMNH | 317648 | Panama | *Molossus* | *coibensis* |
| SMNH | 314300 | Panama | *Molossus* | *coibensis* |
| AMNH | M-18731 | Panama | *Molossus* | *coibensis* |
| AMNH | M-18738 | Panama | *Molossus* | *coibensis* |
| AMNH | M-31432 | Panama | *Molossus* | *coibensis* |
| AMNH | M-63800 | Panama | *Molossus* | *coibensis* |
| AMNH | M-183864 | Panama | *Molossus* | *coibensis* |
| AMNH | M-36669 | Brazil | *Molossus* | *currentium* |
| AMNH | M-23661 | Colombia | *Molossus* | *currentium* |
| AMNH | 34235 | Colombia | *Molossus* | *currentium* |
| AMNH | 34236 | Colombia | *Molossus* | *currentium* |
| UFMS | MA682 | Corumbá - MS | *Molossus* | *currentium* |
| AMNH | M-47213 | Ecuador | *Molossus* | *currentium* |
| AMNH | M-62102 | Ecuador | *Molossus* | *currentium* |
| AMNH | M-62103 | Ecuador | *Molossus* | *currentium* |
| AMNH | M-62104 | Ecuador | *Molossus* | *currentium* |
| TTU | 134662 | Ecuador | *Molossus* | *currentium* |
| TTU | 134664 | Ecuador | *Molossus* | *currentium* |
| TTU | 134665 | Ecuador | *Molossus* | *currentium* |
| AMNH | M-126827 | Honduras | *Molossus* | *currentium* |
| AMNH | M-126856 | Honduras | *Molossus* | *currentium* |
| AMNH | M-41244 | Nicaragua | *Molossus* | *currentium* |
| AMNH | M-212908 | Panama | *Molossus* | *currentium* |
| AMNH | M-178901 | Panama | *Molossus* | *currentium* |
| AMNH | 212908 | Panama | *Molossus* | *currentium* |
| AMNH | 185000 | Panama | *Molossus* | *currentium* |
| AMNH | 184999 | Panama | *Molossus* | *currentium* |
| AMNH | 178900 | Panama | *Molossus* | *currentium* |
| AMNH | 178899 | Panama | *Molossus* | *currentium* |
| AMNH | 31676 | Panama | *Molossus* | *currentium* |
| AMNH | M-184998 | Panama | *Molossus* | *currentium* |
| TTU | 12386 | Paraguya | *Molossus* | *currentium* |
| TTU | 61008 | Paraguay | *Molossus* | *currentium* |
| TTU | 61009 | Paraguay | *Molossus* | *currentium* |
| ROM | 118821 | Ecuador | *Molossus* | *fentoni* |
| ROM | 109176 | Guyana | *Molossus* | *fentoni* |
| ROM | 122583 | Guyana | *Molossus* | *fentoni* |
| ROM | 107869 | Venezuela | *Molossus* | *M. coibensis* |
| AMNH | M-23765 | Cuba | *Molossus* | *milleri* |
| AMNH | M-23766 | Cuba | *Molossus* | *milleri* |
| AMNH | M-186990 | Cuba | *Molossus* | *milleri* |
| AMNH | M-19118 | Cuba | *Molossus* | *milleri* |
| AMNH | M-176097 | Cuba | *Molossus* | *milleri* |
| AMNH | M-176098 | Cuba | *Molossus* | *milleri* |
| AMNH | M-176099 | Cuba | *Molossus* | *milleri* |
| AMNH | MS-4913 | Cuba | *Molossus* | *milleri* |
| AMNH | M-269529 | USA | *Molossus* | *milleri* |
| AMNH | M-269533 | USA | *Molossus* | *milleri* |
| AMNH | M-213934 | Antigua | *Molossus* | *molossus* |
| AMNH | 35951 | Barbados | *Molossus* | *molossus* |
| AMNH | 35953 | Barbados | *Molossus* | *molossus* |
| AMNH | 35952 | Barbados | *Molossus* | *molossus* |
| AMNH | M-213936 | Barbuda | *Molossus* | *molossus* |
| AMNH | M-61762 | Bolivia | *Molossus* | *molossus* |
| AMNH | M-61763 | Bolivia | *Molossus* | *molossus* |
| AMNH | M-246425 | Bolivia | *Molossus* | *molossus* |
| AMNH | M-246426 | Bolivia | *Molossus* | *molossus* |
| MZUSP | 5849 | Brazil | *Molossus* | *molossus* |
| MZUSP | 8231 | Brazil | *Molossus* | *molossus* |
| MZUSP | 15483 | Brazil | *Molossus* | *molossus* |
| MZUSP | 15502 | Brazil | *Molossus* | *molossus* |
| MZUSP | 8348 | Brazil | *Molossus* | *molossus* |
| MZUSP | 8241 | Brazil | *Molossus* | *molossus* |
| MZUSP | 8667 | Brazil | *Molossus* | *molossus* |
| MZUSP | 19460 | Brazil | *Molossus* | *molossus* |
| MZUSP | 8235 | Brazil | *Molossus* | *molossus* |
| MZUSP | 8345 | Brazil | *Molossus* | *molossus* |
| MZUSP | 8671 | Brazil | *Molossus* | *molossus* |
| MZUSP | 8343 | Brazil | *Molossus* | *molossus* |
| MZUSP | 19461 | Brazil | *Molossus* | *molossus* |
| MZUSP | 8327 | Brazil | *Molossus* | *molossus* |
| MZUSP | 8239 | Brazil | *Molossus* | *molossus* |
| MZUSP | 10268 | Brazil | *Molossus* | *molossus* |
| MZUSP | 8347 | Brazil | *Molossus* | *molossus* |
| MZUSP | 8239 | Brazil | *Molossus* | *molossus* |
| MZUSP | 26807 | Brazil | *Molossus* | *molossus* |
| MZUSP | 15509 | Brazil | *Molossus* | *molossus* |
| MZUSP | 15496 | Brazil | *Molossus* | *molossus* |
| MZUSP | 8335 | Brazil | *Molossus* | *molossus* |
| MZUSP | 8244 | Brazil | *Molossus* | *molossus* |
| MZUSP | 10272 | Brazil | *Molossus* | *molossus* |
| MZUSP | 15488 | Brazil | *Molossus* | *molossus* |
| MZUSP | 8673 | Brazil | *Molossus* | *molossus* |
| MZUSP | 15039 | Brazil | *Molossus* | *molossus* |
| MZUSP | 10273 | Brazil | *Molossus* | *molossus* |
| MZUSP | 14899 | Brazil | *Molossus* | *molossus* |
| MZUSP | 14902 | Brazil | *Molossus* | *molossus* |
| MZUSP | 155633 | Brazil | *Molossus* | *molossus* |
| MZUSP | 8344 | Brazil | *Molossus* | *molossus* |
| MZUSP | 27895 | Brazil | *Molossus* | *molossus* |
| MZUSP | 8665 | Brazil | *Molossus* | *molossus* |
| MZUSP | 8656 | Brazil | *Molossus* | *molossus* |
| MZUSP | 8240 | Brazil | *Molossus* | *molossus* |
| MZUSP | 27900 | Brazil | *Molossus* | *molossus* |
| MZUSP | 8228 | Brazil | *Molossus* | *molossus* |
| MZUSP | 27902 | Brazil | *Molossus* | *molossus* |
| MZUSP | 15505 | Brazil | *Molossus* | *molossus* |
| MZUSP | 15577 | Brazil | *Molossus* | *molossus* |
| MZUSP | 10271 | Brazil | *Molossus* | *molossus* |
| MZUSP | 15506 | Brazil | *Molossus* | *molossus* |
| MZUSP | 15484 | Brazil | *Molossus* | *molossus* |
| MZUSP | 15043 | Brazil | *Molossus* | *molossus* |
| MZUSP | 27898 | Brazil | *Molossus* | *molossus* |
| MZUSP | 8232 | Brazil | *Molossus* | *molossus* |
| MZUSP | 8661 | Brazil | *Molossus* | *molossus* |
| MZUSP | 27894 | Brazil | *Molossus* | *molossus* |
| MZUSP | 8353 | Brazil | *Molossus* | *molossus* |
| UNESP | 11493 | Brazil | *Molossus* | *molossus* |
| UNESP | 11448 | Brazil | *Molossus* | *molossus* |
| UNESP | 11457 | Brazil | *Molossus* | *molossus* |
| UNESP | 11894 | Brazil | *Molossus* | *molossus* |
| UNESP | 11896 | Brazil | *Molossus* | *molossus* |
| UNESP | 11898 | Brazil | *Molossus* | *molossus* |
| UNESP | 11895 | Brazil | *Molossus* | *molossus* |
| UNESP | 11900 | Brazil | *Molossus* | *molossus* |
| UNESP | 11901 | Brazil | *Molossus* | *molossus* |
| UNESP | 11916 | Brazil | *Molossus* | *molossus* |
| UNESP | 16457 | Brazil | *Molossus* | *molossus* |
| UNESP | 13273 | Brazil | *Molossus* | *molossus* |
| UNESP | 13268 | Brazil | *Molossus* | *molossus* |
| UNESP | 13270 | Brazil | *Molossus* | *molossus* |
| UNESP | 12787 | Brazil | *Molossus* | *molossus* |
| UNESP | 12786 | Brazil | *Molossus* | *molossus* |
| UNESP | 12911 | Brazil | *Molossus* | *molossus* |
| UNESP | 12783 | Brazil | *Molossus* | *molossus* |
| UNESP | 13178 | Brazil | *Molossus* | *molossus* |
| UNESP | 13190 | Brazil | *Molossus* | *molossus* |
| UNESP | 14565 | Brazil | *Molossus* | *molossus* |
| UNESP | 14566 | Brazil | *Molossus* | *molossus* |
| UNESP | 14570 | Brazil | *Molossus* | *molossus* |
| UNESP | 14568 | Brazil | *Molossus* | *molossus* |
| UNESP | 14567 | Brazil | *Molossus* | *molossus* |
| UNESP | 14569 | Brazil | *Molossus* | *molossus* |
| UNESP | 16456 | Brazil | *Molossus* | *molossus* |
| CMUFLA | 1050 | Brazil | *Molossus* | *molossus* |
| CMUFLA | 1049 | Brazil | *Molossus* | *molossus* |
| CMUFLA | 988 | Brazil | *Molossus* | *molossus* |
| CMUFLA | 536 | Brazil | *Molossus* | *molossus* |
| CMUFLA | 987 | Brazil | *Molossus* | *molossus* |
| CMUFLA | 1052 | Brazil | *Molossus* | *molossus* |
| CMUFLA | 1048 | Brazil | *Molossus* | *molossus* |
| CMUFLA | 356 | Brazil | *Molossus* | *molossus* |
| CMUFLA | 416 | Brazil | *Molossus* | *molossus* |
| CMUFLA | 415 | Brazil | *Molossus* | *molossus* |
| CMUFLA | 408 | Brazil | *Molossus* | *molossus* |
| CMUFLA | 414 | Brazil | *Molossus* | *molossus* |
| CMUFLA | RG13185 | Brazil | *Molossus* | *molossus* |
| CMUFLA | RG1386 | Brazil | *Molossus* | *molossus* |
| CMUFLA | RG1384 | Brazil | *Molossus* | *molossus* |
| CMUFLA | RG13267 | Brazil | *Molossus* | *molossus* |
| CMUFLA | RG13265 | Brazil | *Molossus* | *molossus* |
| PUC | 273 | Brazil | *Molossus* | *molossus* |
| PUC | 274 | Brazil | *Molossus* | *molossus* |
| PUC | 275 | Brazil | *Molossus* | *molossus* |
| PUC | 276 | Brazil | *Molossus* | *molossus* |
| PUC | 277 | Brazil | *Molossus* | *molossus* |
| PUC | 278 | Brazil | *Molossus* | *molossus* |
| PUC | 279 | Brazil | *Molossus* | *molossus* |
| PUC | 280 | Brazil | *Molossus* | *molossus* |
| PUC | 281 | Brazil | *Molossus* | *molossus* |
| PUC | 282 | Brazil | *Molossus* | *molossus* |
| PUC | 284 | Brazil | *Molossus* | *molossus* |
| PUC | 296 | Brazil | *Molossus* | *molossus* |
| PUC | 376 | Brazil | *Molossus* | *molossus* |
| PUC | 29 | Brazil | *Molossus* | *molossus* |
| PUC | 92 | Brazil | *Molossus* | *molossus* |
| PUC | 255 | Brazil | *Molossus* | *molossus* |
| PUC | 272 | Brazil | *Molossus* | *molossus* |
| UFMG | 3402 | Brazil | *Molossus* | *molossus* |
| UFMG | 3403 | Brazil | *Molossus* | *molossus* |
| MZUSP | 386 | Brazil | *Molossus* | *molossus* |
| MZUSP | 17597 | Brazil | *Molossus* | *molossus* |
| MZUSP | 26410 | Brazil | *Molossus* | *molossus* |
| MZUSP | 16866 | Brazil | *Molossus* | *molossus* |
| MZUSP | 16868 | Brazil | *Molossus* | *molossus* |
| MZUSP | 4464 | Brazil | *Molossus* | *molossus* |
| MZUSP | 15048 | Brazil | *Molossus* | *molossus* |
| MZUSP | 15036 | Brazil | *Molossus* | *molossus* |
| MZUSP | 14901 | Brazil | *Molossus* | *molossus* |
| MZUSP | 15046 | Brazil | *Molossus* | *molossus* |
| MZUSP | 15038 | Brazil | *Molossus* | *molossus* |
| MZUSP | 15037 | Brazil | *Molossus* | *molossus* |
| MZUSP | 16849 | Brazil | *Molossus* | *molossus* |
| MZUSP | 16832 | Brazil | *Molossus* | *molossus* |
| MZUSP | 16860 | Brazil | *Molossus* | *molossus* |
| MZUSP | 16845 | Brazil | *Molossus* | *molossus* |
| MZUSP | 16847 | Brazil | *Molossus* | *molossus* |
| MZUSP | 16862 | Brazil | *Molossus* | *molossus* |
| MZUSP | 16853 | Brazil | *Molossus* | *molossus* |
| MZUSP | 16835 | Brazil | *Molossus* | *molossus* |
| MZUSP | 16852 | Brazil | *Molossus* | *molossus* |
| MZUSP | 16854 | Brazil | *Molossus* | *molossus* |
| MZUSP | 4432 | Brazil | *Molossus* | *molossus* |
| MZUSP | 4465 | Brazil | *Molossus* | *molossus* |
| MZUSP | 4443 | Brazil | *Molossus* | *molossus* |
| MZUSP | 16804 | Brazil | *Molossus* | *molossus* |
| MZUSP | 16867 | Brazil | *Molossus* | *molossus* |
| MZUSP | 16865 | Brazil | *Molossus* | *molossus* |
| MZUSP | 16863 | Brazil | *Molossus* | *molossus* |
| MZUSP | 4427 | Brazil | *Molossus* | *molossus* |
| MZUSP | 16792 | Brazil | *Molossus* | *molossus* |
| MZUSP | 16825 | Brazil | *Molossus* | *molossus* |
| MZUSP | 16824 | Brazil | *Molossus* | *molossus* |
| MZUSP | 16822 | Brazil | *Molossus* | *molossus* |
| MZUSP | 16808 | Brazil | *Molossus* | *molossus* |
| MZUSP | 16821 | Brazil | *Molossus* | *molossus* |
| MZUSP | 16818 | Brazil | *Molossus* | *molossus* |
| MZUSP | 16806 | Brazil | *Molossus* | *molossus* |
| MZUSP | 16820 | Brazil | *Molossus* | *molossus* |
| MZUSP | 16823 | Brazil | *Molossus* | *molossus* |
| MZUSP | 16817 | Brazil | *Molossus* | *molossus* |
| MZUSP | 16793 | Brazil | *Molossus* | *molossus* |
| MZUSP | 16807 | Brazil | *Molossus* | *molossus* |
| MZUSP | 4387 | Brazil | *Molossus* | *molossus* |
| MZUSP | 4422 | Brazil | *Molossus* | *molossus* |
| MZUSP | 4407 | Brazil | *Molossus* | *molossus* |
| MZUSP | 4461 | Brazil | *Molossus* | *molossus* |
| MZUSP | 4441 | Brazil | *Molossus* | *molossus* |
| MZUSP | 4447 | Brazil | *Molossus* | *molossus* |
| MZUSP | 4431 | Brazil | *Molossus* | *molossus* |
| MZUSP | 23804 | Brazil | *Molossus* | *molossus* |
| MZUSP | 4386 | Brazil | *Molossus* | *molossus* |
| MZUSP | 4435 | Brazil | *Molossus* | *molossus* |
| MZUSP | 4423 | Brazil | *Molossus* | *molossus* |
| MZUSP | 4462 | Brazil | *Molossus* | *molossus* |
| MZUSP | 21093 | Brazil | *Molossus* | *molossus* |
| MZUSP | 16819 | Brazil | *Molossus* | *molossus* |
| MZUSP | 501 | Brazil | *Molossus* | *molossus* |
| UFMS | MA1275 | Brazil | *Molossus* | *molossus* |
| UFMS | MA1530 | Brazil | *Molossus* | *molossus* |
| UFMS | MA1523 | Brazil | *Molossus* | *molossus* |
| UFMS | MA1548 | Brazil | *Molossus* | *molossus* |
| UFMS | MA699 | Brazil | *Molossus* | *molossus* |
| UFMS | MA1536 | Brazil | *Molossus* | *molossus* |
| UFMS | MA698 | Brazil | *Molossus* | *molossus* |
| UFMS | MA1532 | Brazil | *Molossus* | *molossus* |
| UFMS | ECO271 | Brazil | *Molossus* | *molossus* |
| MNRIO | 3662 | Brazil | *Molossus* | *molossus* |
| MNRIO | 3661 | Brazil | *Molossus* | *molossus* |
| MNRIO | 23064 | Brazil | *Molossus* | *molossus* |
| MNRIO | 23062 | Brazil | *Molossus* | *molossus* |
| MNRIO | 23060 | Brazil | *Molossus* | *molossus* |
| MNRIO | 23058 | Brazil | *Molossus* | *molossus* |
| MNRIO | 23057 | Brazil | *Molossus* | *molossus* |
| MNRIO | 20962 | Brazil | *Molossus* | *molossus* |
| MNRIO | 23065 | Brazil | *Molossus* | *molossus* |
| MNRIO | 30665 | Brazil | *Molossus* | *molossus* |
| MNRIO | 30666 | Brazil | *Molossus* | *molossus* |
| UFMG | 3349 | Brazil | *Molossus* | *molossus* |
| UFMG | 3406 | Brazil | *Molossus* | *molossus* |
| UFRRJ | 6233 | Brazil | *Molossus* | *molossus* |
| UFRRJ | 6229 | Brazil | *Molossus* | *molossus* |
| UFRRJ | 6241 | Brazil | *Molossus* | *molossus* |
| UFRRJ | 6243 | Brazil | *Molossus* | *molossus* |
| UFRRJ | 6242 | Brazil | *Molossus* | *molossus* |
| UFRRJ | 6238 | Brazil | *Molossus* | *molossus* |
| UFMG | 3408 | Brazil | *Molossus* | *molossus* |
| UFRRJ | 4097 | Brazil | *Molossus* | *molossus* |
| UFRRJ | 4051 | Brazil | *Molossus* | *molossus* |
| UFRRJ | 4140 | Brazil | *Molossus* | *molossus* |
| UFRRJ | 4100 | Brazil | *Molossus* | *molossus* |
| UFRRJ | 4019 | Brazil | *Molossus* | *molossus* |
| UFRRJ | 4025 | Brazil | *Molossus* | *molossus* |
| UFRRJ | 6851 | Brazil | *Molossus* | *molossus* |
| UFRRJ | 6878 | Brazil | *Molossus* | *molossus* |
| UFRRJ | 6916 | Brazil | *Molossus* | *molossus* |
| UFRRJ | 1730 | Brazil | *Molossus* | *molossus* |
| UFRRJ | 887 | Brazil | *Molossus* | *molossus* |
| AMNH | 91462 | Brazil | *Molossus* | *molossus* |
| AMNH | 91463 | Brazil | *Molossus* | *molossus* |
| AMNH | 77677 | Brazil | *Molossus* | *molossus* |
| AMNH | 77680 | Brazil | *Molossus* | *molossus* |
| AMNH | 235381 | Brazil | *Molossus* | *molossus* |
| AMNH | 235382 | Brazil | *Molossus* | *molossus* |
| AMNH | 235383 | Brazil | *Molossus* | *molossus* |
| AMNH | 235384 | Brazil | *Molossus* | *molossus* |
| AMNH | 235385 | Brazil | *Molossus* | *molossus* |
| AMNH | 235386 | Brazil | *Molossus* | *molossus* |
| AMNH | 235387 | Brazil | *Molossus* | *molossus* |
| AMNH | 235388 | Brazil | *Molossus* | *molossus* |
| AMNH | 235389 | Brazil | *Molossus* | *molossus* |
| AMNH | 235390 | Brazil | *Molossus* | *molossus* |
| AMNH | 213935 | Codrington village | *Molossus* | *molossus* |
| AMNH | 212569 | Dominica | *Molossus* | *molossus* |
| ROM | 125386 | Dominican Republic | *Molossus* | *molossus* |
| ROM | 125288 | Dominican Republic | *Molossus* | *molossus* |
| ROM | 124287 | Dominican Republic | *Molossus* | *molossus* |
| ROM | 125286 | Dominican Republic | *Molossus* | *molossus* |
| ROM | 125387 | Dominican Republic | *Molossus* | *molossus* |
| AMNH | M-26257 | Ecuador | *Molossus* | *molossus* |
| AMNH | M-267242 | French Guiana | *Molossus* | *molossus* |
| AMNH | M-267244 | French Guiana | *Molossus* | *molossus* |
| AMNH | M-267245 | French Guiana | *Molossus* | *molossus* |
| AMNH | M-267247 | French Guiana | *Molossus* | *molossus* |
| AMNH | M-267248 | French Guiana | *Molossus* | *molossus* |
| AMNH | M-267250 | French Guiana | *Molossus* | *molossus* |
| AMNH | M-267261 | French Guiana | *Molossus* | *molossus* |
| AMNH | M-269102 | French Guiana | *Molossus* | *molossus* |
| ROM | 120848 | Jamaica | *Molossus* | *molossus* |
| ROM | 120849 | Jamaica | *Molossus* | *molossus* |
| ROM | 120847 | Jamaica | *Molossus* | *molossus* |
| ROM | 120820 | Jamaica | *Molossus* | *molossus* |
| ROM | 120819 | Jamaica | *Molossus* | *molossus* |
| ROM | 120797 | Jamaica | *Molossus* | *molossus* |
| ROM | 120796 | Jamaica | *Molossus* | *molossus* |
| ROM | 51458 | Martinique | *Molossus* | *molossus* |
| ROM | 51463 | Martinique | *Molossus* | *molossus* |
| ROM | 51440 | Martinique | *Molossus* | *molossus* |
| ROM | 51462 | Martinique | *Molossus* | *molossus* |
| ROM | 51441 | Martinique | *Molossus* | *molossus* |
| ROM | 51442 | Martinique | *Molossus* | *molossus* |
| ROM | 51444 | Martinique | *Molossus* | *molossus* |
| ROM | 51445 | Martinique | *Molossus* | *molossus* |
| ROM | 51446 | Martinique | *Molossus* | *molossus* |
| ROM | 51448 | Martinique | *Molossus* | *molossus* |
| ROM | 51447 | Martinique | *Molossus* | *molossus* |
| AMNH | M-214096 | Martinique | *Molossus* | *molossus* |
| AMNH | M-30393 | Netherlands Antilles | *Molossus* | *molossus* |
| AMNH | M-213944 | Oranjestad | *Molossus* | *molossus* |
| AMNH | M-213942 | Oranjestad | *Molossus* | *molossus* |
| AMNH | M-234751 | Paraguay | *Molossus* | *molossus* |
| AMNH | M-234752 | Paraguay | *Molossus* | *molossus* |
| AMNH | M-234753 | Paraguay | *Molossus* | *molossus* |
| AMNH | M-234754 | Paraguay | *Molossus* | *molossus* |
| AMNH | M-234762 | Paraguay | *Molossus* | *molossus* |
| AMNH | M-234763 | Paraguay | *Molossus* | *molossus* |
| AMNH | M-234765 | Paraguay | *Molossus* | *molossus* |
| AMNH | M-234767 | Paraguay | *Molossus* | *molossus* |
| AMNH | M-234768 | Paraguay | *Molossus* | *molossus* |
| AMNH | M-248368 | Paraguay | *Molossus* | *molossus* |
| AMNH | M-248369 | Paraguay | *Molossus* | *molossus* |
| AMNH | M-248374 | Paraguay | *Molossus* | *molossus* |
| AMNH | M-248375 | Paraguay | *Molossus* | *molossus* |
| AMNH | M-248376 | Paraguay | *Molossus* | *molossus* |
| AMNH | M-248377 | Paraguay | *Molossus* | *molossus* |
| AMNH | M-248378 | Paraguay | *Molossus* | *molossus* |
| AMNH | M-72360 | Saint Croix | *Molossus* | *molossus* |
| AMNH | M-72361 | Saint Croix | *Molossus* | *molossus* |
| AMNH | M-72362 | Saint Croix | *Molossus* | *molossus* |
| AMNH | M-72363 | Saint Croix | *Molossus* | *molossus* |
| AMNH | M-72365 | Saint John | *Molossus* | *molossus* |
| AMNH | M-72366 | Saint John | *Molossus* | *molossus* |
| AMNH | M-217515 | Saint John | *Molossus* | *molossus* |
| AMNH | M-189421 | Saint John | *Molossus* | *molossus* |
| AMNH | M-72358 | Saint Martin | *Molossus* | *molossus* |
| AMNH | M-214299 | Saint Thomas | *Molossus* | *molossus* |
| AMNH | M-214300 | Saint Thomas | *Molossus* | *molossus* |
| AMNH | M-214302 | Tortola | *Molossus* | *molossus* |
| MNRIO | 23052 | Venezuela | *Molossus* | *molossus* |
| AMNH | M-78437 | Venezuela | *Molossus* | *molossus* |
| AMNH | M-78438 | Venezuela | *Molossus* | *molossus* |
| AMNH | M-78439 | Venezuela | *Molossus* | *molossus* |
| AMNH | M-78444 | Venezuela | *Molossus* | *molossus* |
| AMNH | M-78445 | Venezuela | *Molossus* | *molossus* |
| AMNH | M-78447 | Venezuela | *Molossus* | *molossus* |
| AMNH | M-130629 | Venezuela | *Molossus* | *molossus* |
| AMNH | M-130630 | Venezuela | *Molossus* | *molossus* |
| AMNH | M-131088 | Venezuela | *Molossus* | *molossus* |
| AMNH | M-131089 | Venezuela | *Molossus* | *molossus* |
| AMNH | M-135433 | Venezuela | *Molossus* | *molossus* |
| AMNH | M-135434 | Venezuela | *Molossus* | *molossus* |
| AMNH | M-17020 | Venezuela | *Molossus* | *molossus* |
| AMNH | M-16104 | Venezuela | *Molossus* | *molossus* |
| AMNH | M-16105 | Venezuela | *Molossus* | *molossus* |
| AMNH | M-78451 | Venezuela | *Molossus* | *molossus* |
| AMNH | M-232208 | Uruguay | *Molossus* | *molossus* |
| AMNH | M-232209 | Uruguay | *Molossus* | *molossus* |
| AMNH | M-232210 | Uruguay | *Molossus* | *molossus* |
| AMNH | M-232211 | Uruguay | *Molossus* | *molossus* |
| AMNH | M-248392 | Paraguay | *Molossus* | *molossus* |
| TTU | 12555 | Costa Rica | *Molossus* | *pretiosus* |
| TTU | 12565 | Costa Rica | *Molossus* | *pretiosus* |
| UFRRJ | 6896 | Jaíba, MG | *Molossus* | *pretiosus* |
| UFRRJ | 6875 | Jaíba, MG | *Molossus* | *pretiosus* |
| UFRRJ | 6946 | Jaíba, MG | *Molossus* | *pretiosus* |
| UFRRJ | 6943 | Jaíba, MG | *Molossus* | *pretiosus* |
| UFRRJ | 6963 | Jaíba, MG | *Molossus* | *pretiosus* |
| UFRRJ | 6945 | Jaíba, MG | *Molossus* | *pretiosus* |
| UFRRJ | 6912 | Jaíba, MG | *Molossus* | *pretiosus* |
| UFRRJ | 6911 | Jaíba, MG | *Molossus* | *pretiosus* |
| UFRRJ | 6914 | Jaíba, MG | *Molossus* | *pretiosus* |
| UFRRJ | 6944 | Jaíba, MG | *Molossus* | *pretiosus* |
| UFRRJ | 6915 | Jaíba, MG | *Molossus* | *pretiosus* |
| TTU | 12342 | Nicaragua | *Molossus* | *pretiosus* |
| TTU | 12343 | Nicaragua | *Molossus* | *pretiosus* |
| CMUFLA |  | Sao Jose do Rio Preto - SP | *Molossus* | *pretiosus* |
| AMNH | M-16076 | Venezuela | *Molossus* | *pretiosus* |
| AMNH | M-16077 | Venezuela | *Molossus* | *pretiosus* |
| AMNH | M-16078 | Venezuela | *Molossus* | *pretiosus* |
| AMNH | M-16079 | Venezuela | *Molossus* | *pretiosus* |
| AMNH | M-16080 | Venezuela | *Molossus* | *pretiosus* |
| AMNH | M-16081 | Venezuela | *Molossus* | *pretiosus* |
| AMNH | M-16084 | Venezuela | *Molossus* | *pretiosus* |
| AMNH | M-17042 | Venezuela | *Molossus* | *pretiosus* |
| AMNH | M-23780 | Venezuela | *Molossus* | *pretiosus* |
| AMNH | M-23781 | Venezuela | *Molossus* | *pretiosus* |
| AMNH | 17037 | Venezuela, Juapure | *Molossus* | *pretiosus* |
| AMNH | 17039 | Venezuela, Juapure | *Molossus* | *pretiosus* |
| AMNH | 17040 | Venezuela, Juapure | *Molossus* | *pretiosus* |
| AMNH | 17041 | Venezuela, Juapure | *Molossus* | *pretiosus* |
| AMNH | 17042 | Venezuela, Juapure | *Molossus* | *pretiosus* |
| AMNH | 17043 | Venezuela, Juapure | *Molossus* | *pretiosus* |
| SMNH | 303852 | Venezuela, São Francisco de Cara | *Molossus* | *pretiosus* |
| SMNH | 143832 | Venezuela. Macuto | *Molossus* | *pretiosus* |
| SMNH | 102780 | Venezuela: Destrito Federal | *Molossus* | *pretiosus* |
| SMNH | 102749 | Venezuela: La Guaira | *Molossus* | *pretiosus* |
| SMNH | 102745 | Venezuela: La Guaira | *Molossus* | *pretiosus* |
| SMNH | 102744 | Venezuela: La Guaira | *Molossus* | *pretiosus* |
| SMNH | 102748 | Venezuela: La Guaira | *Molossus* | *pretiosus* |
| SMNH | 102767 | Venezuela: Macuto | *Molossus* | *pretiosus* |
| SMNH | 102768 | Venezuela: Macuto | *Molossus* | *pretiosus* |
| SMNH | 143833 | Venezuela: Macuto | *Molossus* | *pretiosus* |
| SMNH | 143835 | Venezuela: Macuto | *Molossus* | *pretiosus* |
| SMNH | 143834 | Venezuela: Macuto | *Molossus* | *pretiosus* |
| SMNH | 102747 | Venezuela:La Guaira | *Molossus* | *pretiosus* |
| AMNH | M-261852 | Bolivia | *Molossus* | *rufus* |
| AMNH | M-263285 | Bolivia | *Molossus* | *rufus* |
| AMNH | M-211279 | Bolivia | *Molossus* | *rufus* |
| CMUFLA | 10745 | Brazil | *Molossus* | *rufus* |
| CMUFLA | 979 | Brazil | *Molossus* | *rufus* |
| UNESP | 15988 | Brazil | *Molossus* | *rufus* |
| UNESP | 15990 | Brazil | *Molossus* | *rufus* |
| UNESP | 15989 | Brazil | *Molossus* | *rufus* |
| UNESP | 16478 | Brazil | *Molossus* | *rufus* |
| UNESP | 16477 | Brazil | *Molossus* | *rufus* |
| UNESP | 16474 | Brazil | *Molossus* | *rufus* |
| UNESP | 11493 | Brazil | *Molossus* | *rufus* |
| UNESP | 10453 | Brazil | *Molossus* | *rufus* |
| UNESP | 10439 | Brazil | *Molossus* | *rufus* |
| UNESP | 10449 | Brazil | *Molossus* | *rufus* |
| UNESP | 10447 | Brazil | *Molossus* | *rufus* |
| UNESP | 10747 | Brazil | *Molossus* | *rufus* |
| UNESP | 10748 | Brazil | *Molossus* | *rufus* |
| UNESP | 10749 | Brazil | *Molossus* | *rufus* |
| UNESP | 10745 | Brazil | *Molossus* | *rufus* |
| UNESP | 10767 | Brazil | *Molossus* | *rufus* |
| UNESP | 10766 | Brazil | *Molossus* | *rufus* |
| UNESP | 11917 | Brazil | *Molossus* | *rufus* |
| UNESP | 11918 | Brazil | *Molossus* | *rufus* |
| UNESP | 11923 | Brazil | *Molossus* | *rufus* |
| UNESP | 11925 | Brazil | *Molossus* | *rufus* |
| UNESP | 11919 | Brazil | *Molossus* | *rufus* |
| UNESP | 12482 | Brazil | *Molossus* | *rufus* |
| UNESP | 10735 | Brazil | *Molossus* | *rufus* |
| UNESP | 10736 | Brazil | *Molossus* | *rufus* |
| UNESP | 10740 | Brazil | *Molossus* | *rufus* |
| CMUFLA | 979 | Brazil | *Molossus* | *rufus* |
| MZUSP | 1489 | Brazil | *Molossus* | *rufus* |
| MZUSP | 21036 | Brazil | *Molossus* | *rufus* |
| MZUSP | 21083 | Brazil | *Molossus* | *rufus* |
| MZUSP | 21085 | Brazil | *Molossus* | *rufus* |
| MZUSP | 17595 | Brazil | *Molossus* | *rufus* |
| MZUSP | 4381 | Brazil | *Molossus* | *rufus* |
| MZUSP | 4439 | Brazil | *Molossus* | *rufus* |
| MZUSP | 4450 | Brazil | *Molossus* | *rufus* |
| MZUSP | 4428 | Brazil | *Molossus* | *rufus* |
| MZUSP | 4391 | Brazil | *Molossus* | *rufus* |
| MZUSP | 5630 | Brazil | *Molossus* | *rufus* |
| MZUSP | 4463 | Brazil | *Molossus* | *rufus* |
| MZUSP | 4437 | Brazil | *Molossus* | *rufus* |
| MZUSP | 8729 | Brazil | *Molossus* | *rufus* |
| UFMS | MA1318 | Brazil | *Molossus* | *rufus* |
| MNRIO | 3996 | Brazil | *Molossus* | *rufus* |
| MNRIO | 3296 | Brazil | *Molossus* | *rufus* |
| MNRIO | 3505 | Brazil | *Molossus* | *rufus* |
| UFMS | MA649 | Brazil | *Molossus* | *rufus* |
| UFMS | MA1369 | Brazil | *Molossus* | *rufus* |
| MNRIO | 3603 | Brazil | *Molossus* | *rufus* |
| UFRRJ | 6232 | Brazil | *Molossus* | *rufus* |
| UFRRJ | 6228 | Brazil | *Molossus* | *rufus* |
| UFRRJ | 6302 | Brazil | *Molossus* | *rufus* |
| UFRRJ | 6231 | Brazil | *Molossus* | *rufus* |
| UFRRJ | 6299 | Brazil | *Molossus* | *rufus* |
| UFRRJ | 6301 | Brazil | *Molossus* | *rufus* |
| UFRRJ | 6229 | Brazil | *Molossus* | *rufus* |
| MNRIO | 11198 | Brazil | *Molossus* | *rufus* |
| UFMS | MA1360 | Brazil | *Molossus* | *rufus* |
| UFMS | MA1374 | Brazil | *Molossus* | *rufus* |
| AMNH | 77669 | Brazil | *Molossus* | *rufus* |
| AMNH | 77670 | Brazil | *Molossus* | *rufus* |
| AMNH | 77671 | Brazil | *Molossus* | *rufus* |
| AMNH | 77673 | Brazil | *Molossus* | *rufus* |
| AMNH | 77674 | Brazil | *Molossus* | *rufus* |
| AMNH | 77675 | Brazil | *Molossus* | *rufus* |
| SMNH | 393793 | Brazil | *Molossus* | *rufus* |
| SMNH | 393791 | Brazil | *Molossus* | *rufus* |
| SMNH | 393792 | Brazil | *Molossus* | *rufus* |
| SMNH | 393790 | Brazil | *Molossus* | *rufus* |
| SMNH | 393789 | Brazil | *Molossus* | *rufus* |
| SMNH | 393788 | Brazil | *Molossus* | *rufus* |
| SMNH | 393787 | Brazil | *Molossus* | *rufus* |
| SMNH | 393786 | Brazil | *Molossus* | *rufus* |
| AMNH | 267263 | French Guiana | *Molossus* | *rufus* |
| AMNH | 267264 | French Guiana | *Molossus* | *rufus* |
| AMNH | 267267 | French Guiana | *Molossus* | *rufus* |
| AMNH | 267268 | French Guiana | *Molossus* | *rufus* |
| AMNH | 26270 | French Guiana | *Molossus* | *rufus* |
| AMNH | 267539 | French Guiana | *Molossus* | *rufus* |
| AMNH | M-123306 | Honduras | *Molossus* | *rufus* |
| AMNH | M-123307 | Honduras | *Molossus* | *rufus* |
| AMNH | M-123308 | Honduras | *Molossus* | *rufus* |
| AMNH | M-123309 | Honduras | *Molossus* | *rufus* |
| AMNH | M-123310 | Honduras | *Molossus* | *rufus* |
| AMNH | M-123317 | Honduras | *Molossus* | *rufus* |
| AMNH | M-145150 | Mexico | *Molossus* | *rufus* |
| AMNH | M-178743 | Mexico | *Molossus* | *rufus* |
| AMNH | M-190184 | Mexico | *Molossus* | *rufus* |
| AMNH | M-190185 | Mexico | *Molossus* | *rufus* |
| AMNH | M-203938 | Mexico | *Molossus* | *rufus* |
| AMNH | M-203939 | Mexico | *Molossus* | *rufus* |
| AMNH | M-203940 | Mexico | *Molossus* | *rufus* |
| AMNH | M-203941 | Mexico | *Molossus* | *rufus* |
| AMNH | M-203942 | Mexico | *Molossus* | *rufus* |
| AMNH | M-239241 | Mexico | *Molossus* | *rufus* |
| AMNH | M-239242 | Mexico | *Molossus* | *rufus* |
| AMNH | M-209240 | Paraguay | *Molossus* | *rufus* |
| AMNH | M0239249 | Paraguay | *Molossus* | *rufus* |
| AMNH | M-217570 | Paraguay | *Molossus* | *rufus* |
| AMNH | M-217571 | Paraguay | *Molossus* | *rufus* |
| AMNH | M-217572 | Paraguay | *Molossus* | *rufus* |
| AMNH | M-248367 | Paraguay | *Molossus* | *rufus* |
| SMNH | 559802 | Mexico | *Molossus* | *sinaloae* |
| SMNH | 508997 | Mexico | *Molossus* | *sinaloae* |
| SMNH | 509001 | Mexico | *Molossus* | *sinaloae* |
| SMNH | 508996 | Mexico | *Molossus* | *sinaloae* |
| SMNH | 508998 | Mexico | *Molossus* | *sinaloae* |
| AMNH | M-24524 | Mexico | *Molossus* | *sinaloae* |
| ACUNHC | 279 | Venezuela | *Molossus* | *sp* |
| ACUNHC | 261 | Venezuela | *Molossus* | *sp* |
| ROM | 125388 | Dominican Republic | *Molossus* | *verrilli* |
| ROM | 125289 | Dominican Republic | *Molossus* | *verrilli* |
| ROM | 125385 | Dominican Republic | *Molossus* | *verrilli* |
| AMNH | M-25764 | Dominican Republic | *Molossus* | *verrilli* |
| ROM | 31781 | Dominican Republic | *Molossus* | *verrilli* |
| ROM | 31782 | Dominican Republic | *Molossus* | *verrilli* |
| ROM | 125289 | Dominican Republic | *Molossus* | *verrilli* |
| AMNH | M-236706 | Haiti | *Molossus* | *verrilli* |
| AMNH | M-236707 | Haiti | *Molossus* | *verrilli* |
| AMNH | M-236708 | Haiti | *Molossus* | *verrilli* |
| AMNH | M-236714 | Haiti | *Molossus* | *verrilli* |
